# Real-Time Collection of Conidia Released from Living Single Colonies of *Podosphaera aphanis* on Strawberry Leaves under Natural Conditions with Electrostatic Techniques

**DOI:** 10.3390/plants11243453

**Published:** 2022-12-09

**Authors:** Shuka Ayabe, Yutaka Kimura, Naoki Umei, Yoshihiro Takikawa, Koji Kakutani, Yoshinori Matsuda, Teruo Nonomura

**Affiliations:** 1 Department of Agriculture Science, Faculty of Agriculture, Kindai University, Nara 631-8505, Japan; 2 Plant Center, Institute of Advanced Technology, Kindai University, Wakayama 642-0017, Japan; 3 Pharmaceutical Research and Technology Institute and Anti-Aging Centers, Kindai University, Osaka 577-8502, Japan; 4 Agricultural Technology and Innovation Research Institute, Kindai University, Nara 631-8505, Japan

**Keywords:** catenated conidia, conidiophores, electrostatic spore collectors, lifelong production, powdery mildews, strawberry plants

## Abstract

Powdery mildew fungi produce progeny conidia on conidiophores, and promote the spread of powdery mildew diseases by dispersal of the conidia from conidiophores in the natural environment. To gain insights and devise strategies for preventing the spread of powdery mildew infection, it is important to clarify the ecological mechanism of conidial dispersal from conidiophores. In this study, all of the progeny conidia released from single colonies of strawberry powdery mildew fungus (*Podosphaera aphanis* (Wallroth) U. Braun and S. Takamatsu var. *aphanis* KSP-7N) on true leaves of living strawberry plants (*Fragaria* × *ananassa* Duchesne ex Rozier cv. Sagahonoka) were consecutively collected over the lifetime of the colony with an electrostatic rotational spore collector (insulator drum) under greenhouse conditions, and counted under a high-fidelity digital microscope. The insulator drum consisted of a round plastic container, copper film, thin and transparent collector film, electrostatic voltage generator, and timer mechanism. When negative charge was supplied from the voltage generator to the copper film, the collector film created an attractive force to trap conidia. The electrostatically activated collector film successfully attracted progeny conidia released from the colony. Experiment was carried out at just one colony on one leaf for each month (in February, May, July, October, November, and December in 2021), respectively. Each collector film was exchanged for a new collector film at 24 h intervals until KSP-7N ceased to release progeny conidia from single colonies. Collection experiments were carried out to estimate the total number of conidia released from a single KSP-7N colony over a 35–45-day period after inoculation. During the fungal lifetime, KSP-7N released an average of 6.7 × 10^4^ conidia from each of the single colonies at approximately 816 h. In addition, conidial release from KSP-7N colonies was largely affected by the light intensity and day length throughout a year; the number of conidia released from single KSP-7N colonies in night-time was clearly smaller than that in daytime, and the time of conidial release from single KSP-7N colonies was shorter by approximately 2 to 4 h in autumn and winter than in spring and summer. The ecological characteristics related to conidial releases from KSP-7N colonies will be helpful information for us to successfully suppress the spread of strawberry powdery mildews onto host plants under greenhouse conditions.

## 1. Introduction

Powdery mildew caused by the obligate fungus *Podosphaera aphanis* U. Braun and S. Takamatsu var. *aphanis* (syn. *Sphaerotheca humuli* Burrill or *S. macularis* Jaczewski f. sp. *fragariae*) [1,2] is one of the most dangerous diseases of strawberry plants of the Rosaceae family in many countries, and tends to affect the epigeal organs (i.e., leaves, petioles, stolons, receptacles, flowers, fruits, and runners) [3,4,5,6,7]. Ultimately, strawberry fruit production is significantly reduced, and the quality deteriorates rapidly with severe infection [6,7,8,9,10]. In Japan, farmers begin to plant the strawberry seedlings, purchased from a seedling company, from September to November, and then harvest the strawberry fruits from December to April. On the other hand, powdery mildews occur during the growing stages from May to June, after plantings from September to November, and during the harvest periods from March to April [11,12,13]. Thus, the strawberry plants are highly susceptible to powdery mildew diseases in cooler seasons from September to November, and from March to April. Recently, strawberry powdery mildew fungi have appeared on the true leaves of strawberry plants (*Fragaria* × *ananassa* Duchesne ex Rozier, cv. Sagahonoka) highly grown in our greenhouses [14]. After a conidium from the powdery mildew-infected strawberry leaves was isolated and the fungi were multiplied, we identified the isolate as strawberry powdery mildew fungi on the basis of previously described morphological characteristics [2,13,15] and ribosomal DNA internal transcribed spacer (rDNA-ITS) sequences [16], and designated the isolate *Po. aphanis* (Wallroth) U. Braun and S. Takamatsu var. *aphanis* KSP-7N in our previous study [14]; in addition, the KSP-7N isolate was highly pathogenic to the commercial cultivars (*Fragaria* × *ananassa* Duchesne) of strawberry plants, wild strawberry (*F. vesca* L.) and false strawberry (*Potentilla hebiichigo*) (syn. *Duchesnea chrysantha* Miq.) [14].

In general, to control powdery mildew disease on strawberry plants, physical [17,18], chemical [11,12,19,20], and biological [21,22,23,24,25] methods have been applied, in Japan and elsewhere. The main objective of our study was to efficiently control powdery mildew disease without using agricultural chemicals. The pathogens form asexual progeny conidia on conidiophores, which are an infection source of host plants and can lead to further dissemination of powdery mildews over a wide area by dispersal of progeny conidia from conidiophores under natural conditions [26,27,28]. To prevent the spread of powdery mildew infection, from an ecological point of view, it is very important to confirm the conidial dispersal from the conidiophores. Therefore, using a high-fidelity digital microscope, the conidiogenesis of several powdery mildew pathogens on leaves of host plants were carefully examined; this allowed us to observe the developments of powdery mildew pathogens on host leaves without fixing or staining, using *Pseudoidium neolycopersici* L. Kiss [29,30], *Blumeria graminis* f. sp. *hordei* Marchal race 1 [31], *Po. xanthii* (Schlechtend.:Fr.) Pollacci [32] and *Po. aphanis* (Wallroth) U. Braun and S. Takamatsu var. *aphanis* [14]. Regarding the strawberry powdery mildew fungi, Iwasaki et al. [14] recently confirmed that the KSP-7N isolate forms catenated conidia consisting of a maximum of six immature conidia, one undivided conidial cell (the upper cell), one generative cell (gc), and one basal cell (bc), and that the first matured conidium and subsequent matured conidia are dispersed from the conidiophores via division of the upper cell and septation in the gc, respectively, with initial conidiophore formation (completion of a set of developed cells). However, subsequent conidial releases showed that KSP-7N conidiophores repeatedly released progeny conidia with gradually elongating the gc in the conidiophore, and eventually became longer than the melon powdery mildew conidiophores (*Po. xanthii* KMP-6N) as previously described by Iwasaki et al. [14]. We also demonstrated that a conidiophore (approximately 400-μm in length) of *Po. aphanis* KSP-7N formed an average of 38 conidia during its lifetime of approximately 96 h under a natural condition. Based on these results, we were largely interested in confirming when and how many conidia are formed and dispersed from single strawberry powdery mildew colonies on host leaves. Therefore, in the present study, we attempted to quantitatively analyse the lifetime production of asexual progeny conidia released from single strawberry powdery mildew colonies each month, irrespective of whether it was day or night, under greenhouse conditions.

To successfully collect progeny conidia from single colonies of some powdery mildew fungi throughout their lifetime, we utilised electrostatic techniques. Matsuda et al. [33] previously described that conidia that enter into the electric field are attracted to polarised dipole insulators, and then trapped on them. From the principles of electrostatics, electrostatic spore collectors using a dielectrically polarised insulator plate, probe, or drum were devised, and all progeny conidia formed from living single powdery mildew colonies on barley (*B. graminis* f. sp. *hordei*) [34], tomato (*Ps. neolycopersici*) [35] and melon (*Po. xanthii*) [36] were efficiently and successfully collected throughout their lifetime. In the present study, we used a dielectrically polarised insulator drum (an electrostatic rotational spore collector, i.e., a rotary drum) to collect all conidia released during the growth of single KSP-7N colonies on true leaves of living strawberry seedlings under greenhouse conditions, and counted all conidia attracted to the insulators (i.e., insulator films wound around the insulator drum) during the lifetimes of the colonies with a digital microscope. This electrostatic technique allowed us to successfully collect matured conidia from single colonies without harming the powdery mildew conidia, and previously clarify the optimised electrostatic conditions for attracting conidia with melon powdery mildew KMP-6N isolates [36]. To our knowledge, this is the first study quantitatively analysing the lifelong production of progeny conidia released from single strawberry powdery mildew colonies (*Po. aphanis*) on living leaves of host seedlings. This scientific information will be essential for comprehending the ecological mechanism underlying the expansion of powdery mildew infection under greenhouse conditions, and will aid the development and adoption of eco-friendly preventive measures against the spread of powdery mildew fungi.

## 2. Results

### 2.1. Morphological Observation of KSP-7N Conidia and Conidiophores Developed under Static Electricity

**Figure 1:**
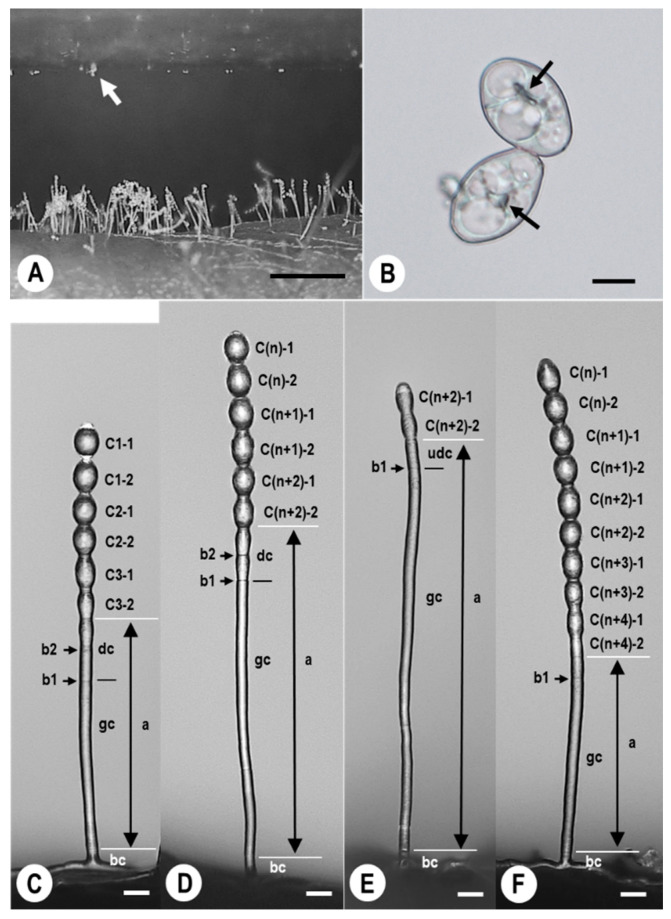
Micrographs of conidia collected from a single KSP-7N powdery mildew colony to the insulator film with an electrostatic technique (**A**,**B**), and conidiophores observed during electrostatic spore collection under greenhouse conditions (**C**–**E**) or in darkness (**F**). (**A**) Digital micrograph revealing the collection of progeny conidia dispersed from a single 10-day-old KSP-7N powdery mildew colony on a strawberry leaf to an insulator film. The film (arrowed) charged at 1.0 nC was placed 900 μm from the top of the KSP-7N conidiophore. The scale bar is 600 μm. (**B**) Light micrograph revealing conidia attracted on the film. Oil and fibrosin bodies (arrows) were observed in single KSP-7N conidia. The scale bar is 10 μm. (**C**–**F**) Digital micrographs revealing conidiophores possessing six conidial cells (C1-1 to C3-2) in full-length chains, a divided conidia cell (dc), a generative cell (gc), and a basal cell (bc) on hyphae in KSP-7N colonies (**C**), six conidial cells (C(n)−1 to C(n + 2)−2), dc, gc, and bc (**D**), immature conidial cells (C(n + 2)−1, C(n + 2)−2), an undivided conidial cell (udc), gc, and bc (**E**) under greenhouse conditions, and ten conidial cells (C(n + 1)−1 to C(n + 4)−2), dc, gc, and bc (**F**) in darkness. Conidial cells were produced by repeatedly elongating the gc and dividing twice via septation (black right-pointing b1 and b2 arrows). The double-pointed arrow (a) indicates the length of an elongated gc. Once the septum between the apical conidium and the next conidial cell in (**C**,**D**) had fully constricted, KSP-7N conidiophores released progeny conidia. The scale bars are 20 μm.

Progeny conidia dispersed from conidiophores in a single KSP-7N colony were electrostatically collected to the negatively polarised insulator film (Figure 1A). The sizes and shapes of conidia collected were 28.3 ± 2.3 × 19.8 ± 1.4 μm, and the shapes were ellipsoid-ovoid to doliiform-limoniform, respectively. They contained oil and fibrosin bodies, as observed using BX-60 light microscope (BX-60 LM instrument) (Figure 1B). Original conidiophores formed in the colonies were catenated, with a maximum of six conidia (C1-1 to C3-2) forming in straight chains, a divided conidial cell (dc), a gc, and a bc on hyphae (revealed by KH-2700 high-fidelity digital microscope (KH-2700 DM instrument)) (Figure 1C). Figure 1C–E shows the developmental process of KSP-7N conidiophores. The lengths of conidiophores (Figure 1D,E) in single colonies clearly exceeded those of the original conidiophores (Figure 1C) due to release the conidia with gradually upwards elongating the gc in conidiophores. The gc lengths in the conidiophores were 184.0 ± 28.6 × 9.8 ± 1.1 μm (Figure 1C), 268.3 ± 16.2 × 9.8 ± 1.1 μm (Figure 1D) and 319.5 ± 44.8 × 9.8 ± 1.1 μm (Figure 1E). Moreover, as the single colonies developed, the numbers of conidiophores shown in Figure 1D increased during electrostatic spore collection. Ultimately, the numbers of conidiophores shown in Figure 1E increased at the end of the electrostatic spore collection. On the other hand, KSP-7N mostly produced a maximum of six conidia in chains on each conidiophore, irrespective of conidiophore lengths (gc lengths) during the daytime, whereas KSP-7N formed continuous chains (7–10 conidia on conidiophores) without fully formed constrictions between the conidia at the top of the conidiophores, in darkness (night-time) over 12 h (Figure 1F).

### 2.2. Germination Rates of KSP-7N Conidia Electrostatically Collected on Insulator Films

After the KSP-7N conidia were electrostatically collected from a single KSP-7N colony for each of the six months of experiment, the germination rates were measured, and the means and standard deviations (SD) of five replicates (collected on the 1st, 6th, 11th, 16th, and 21st films) were calculated. The conidial germination rates were 86.5 ± 0.8, 85.5 ± 0.6, 82.4 ± 0.1, 84.5 ± 0.2, 85.0 ± 0.5, and 86.2 ± 0.4% in February, May, July, October, November, and December, respectively.

### 2.3. Total Number of Progeny Conidia Electrostatically Collected from Single KSP-7N Colonies under Greenhouse Conditions

**Figure 2:**
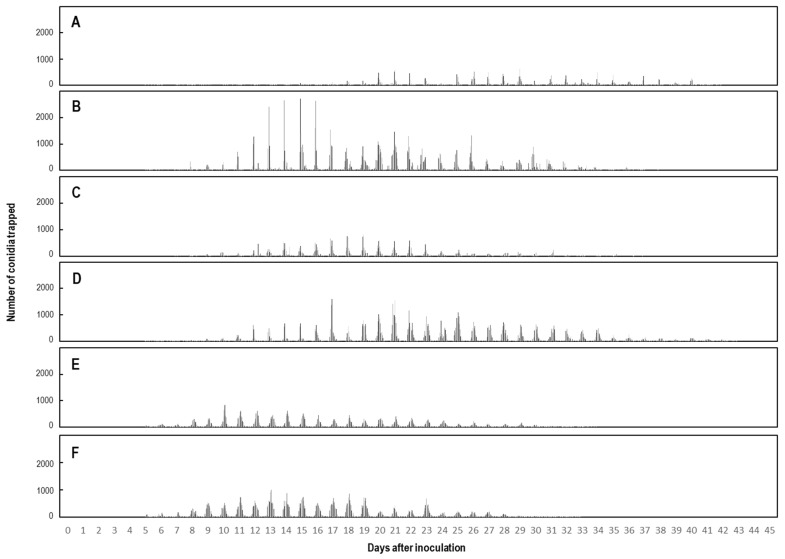
Lifelong production of asexual conidia released from single KSP-7N colonies was assessed from total number of progeny conidia collected in 1 h. Progeny conidia from single KSP-7N colonies on leaves of living strawberry seedlings were trapped using an electrostatic rotational spore collector (insulator drum) in February (**A**), May (**B**), July (**C**), October (**D**), November (**E**), and December (**F**) in 2021. In each panel, (**A**–**F**) was recorded 45 days, which is more than 1 month.

**Figure 3:**
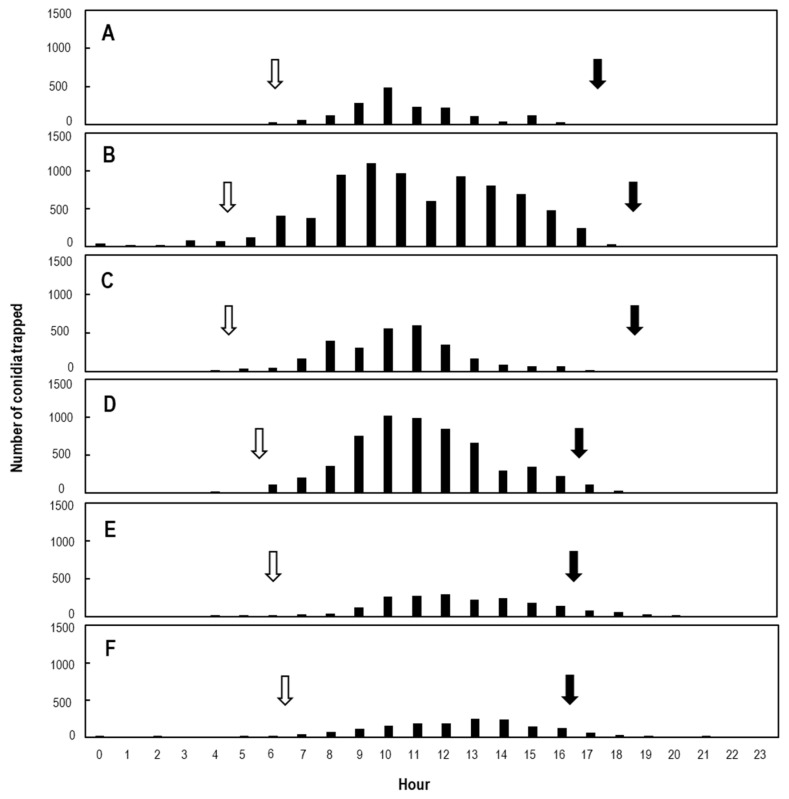
Time periods of conidial releases were shorter by ca. 3.0 h in December than in July. Mature conidia from single KSP-7N colonies on leaves of living strawberry seedlings at 20 days after inoculation were trapped over a period of 24 h with an electrostatic spore collector (insulator drum) in February (**A**), May (**B**), July (**C**), October (**D**), November (**E**), and December (**F**) in 2021. The data were represented in 1 h periods. Open and closed arrows indicated the times of sunrise and sunset each month, respectively.

The production of all progeny conidia collected from six single KSP-7N colonies throughout their lifetime was assessed from total number of conidia collected in 1 h (Figure 2). Asexual progeny conidia were first collected from 5-day-old KSP-7N colonies with a dielectrically polarised insulator drum, and were then collected for a further ca. 4–6 weeks from all colonies. The expansion of colonies stopped 14–15 days after inoculation. However, progeny conidia were continuously released from the KSP-7N colonies thereafter for 14–24 days (ca. 2–3 weeks) (Figure 2). The colony areas, numbers of conidiophores in single KSP-7N colonies, duration of conidial secession, and total number of progeny conidia released by individual KSP-7N colonies throughout their lifetime are shown in Table 1, respectively. The numbers of progeny conidia released were between 2.8 × 10^4^ and 10.3 × 10^4^ conidia per colony, with an average of 6.7 × 10^4^ conidia released during the lifespan of ca. 816 h. Next, based on the collected data, the number of conidia released and time periods of conidial releases from individual 20-day-old colonies in a single day were compared (Figure 3). Table 2 shows the dates of sunrise and sunset, and periods of daytime and night-time, for each month when the electrostatic spore collection was conducted with 20-day-old colonies (as shown in Figure 3). As a whole, the time periods of releases of conidia were longer by ca. 3.0 h in July than December. Moreover, Table 3 shows the total number of conidia released from single 20-day-old KSP-7N colonies in a single day during the daytime versus at night-time for each month (20 February, 20 May, 20 July, 20 October, 20 November, and 20 December 2021). There was a definite difference between the number of progeny conidia released during the daytime versus at night-time for each month, as shown in Table 3. Thus, the number of progeny conidia released during the daytime was clearly larger than that during the night-time, with statistical analysis.

## 3. Discussion

Recently, Iwasaki et al. [14] identified powdery mildew Japanese isolates that appeared on the leaves of commercial strawberries (cv. Sagahonoka) in greenhouses as *Po. aphanis* (Wallroh) var. *aphanis* KSP-7N, based on morphological characteristics of the fungi and genetic analysis of the rDNA-ITS region. The morphological characteristics of KSP-7N isolate were very similar to those of domestic and foreign isolates that we previously subjected to microscopic analyses [2,5,13,15,37,38,39,40,41,42,43,44]. Additionally, in host range analyses, Iwasaki et al. [14] confirmed that the KSP-7N isolate heavily infected and sporulated inoculated leaves of all tested commercial strawberries of the cultivar *Fragaria* × *ananassa* Duchesne, as previously reported [3,4,45,46,47].

Powdery mildew greatly promotes the spread of infection by releasing progeny conidia from conidiophores in fungal colonies [28,48,49]. Therefore, from ecological and epidemiological perspectives, we mainly focused on estimating when, how, and how many asexual progeny conidia are dispersed from single powdery mildew colonies found on host plants throughout their lifetime, using the electrostatic techniques described in the present study. Regarding the fundamental principles of static electricity, Leach [50] reported that fungal spores dispersed by strong wind become charged electrically at the moment of release. In addition, Griffith [51] and Halliday et al. [52] reported that the dielectrically polarised insulators create a non-uniform electric field around themselves, which gives rise to an electrostatic force. Moreover, in our previous studies [33,53], we reported that both negatively and positively polarised insulators (insulator cylinders) can successfully attract the powdery mildew conidia by exploiting electrostatic force. After that, we devised some novel apparatuses to attract fungal conidia using the electrostatic force, and counted all progeny conidia released from the living single colonies of powdery mildew fungi on host leaves using negatively polarised insulator plates [34], probes [35], and drums [36] throughout the fungal lifetime. Fortunately, the powdery mildew isolates grew on host leaves, and formed living conidia from the conidiophores, even when exposed to an electrostatic force over their lifetime. In addition, dielectrically polarised insulators detached only the conidia at the top of conidiophores that caused constriction between conidial cells on conidiophores in their colonies [48,54]. Moreover, the conidia collected on the insulators maintained their high germinative capacity [35,36]. Among the powdery mildew isolates that we tested on previous works using the apparatuses mentioned above, powdery mildew fungus of barley (*B. graminis* f. sp. *hordei* KBP-01; catenated conidia) released ca. 1.2 × 10^5^ conidia from a single colony [34], powdery mildew fungus of tomato (*Ps. neolycopersici* KTP-01; non-catenated conidia) released ca. 5 × 10^3^ conidia [35], and powdery mildew fungus of melon (*Po. xanthii* KMP-6N; catenated conidia) released ca. 1.26 × 10^5^ conidia during its lifespan [36]. Thus, the electrostatic techniques enabled us to collect all progeny conidia dispersed from living single powdery mildew colonies throughout their lifetime. In the present study, for the first time, we clarified the total number of progeny conidia released from living single KSP-7N colonies on strawberry leaves during their lifetime with an electrostatically activated insulator film (a rotary drum), because conidia release from KSP-7N has not previously been monitored or analysed in detail. Barley, melon and strawberry powdery mildew Japanese isolates produced catenate conidia on conidiophores; KBP-01 produced chains of a maximum of eight conidia per conidiophore [31], whereas KMP-6N and KSP-7N formed chains of a maximum of six conidia per conidiophore, respectively [14,32]. Ultimately, the numbers of progeny conidia released from single KSP-7N colonies were somewhat different from those released from single KBP-01 or KMP-6N colonies, although continuance (h) of conidial secession and the total number of conidia released from single KSP-7N conidiophores were the same as from single KBP-01 and KMP-6N conidiophores [14,31,32]. Therefore, we compared the colony areas of and number of conidiophores in single KSP-7N colonies with those in single KMP-6N colonies (belonging to the same *Podosphaera* genus), after conidial release from fungal colonies had ceased. The colony areas of single KSP-7N colonies were smaller than those of single KMP-6N colonies (average 1.8 cm^2^; [36]). In addition, there were fewer conidiophores in single KSP-7N colonies than single KMP-6N colonies (average of 1211 conidiophores; [36]). Ultimately, the lifelong production of mature conidia from single KSP-7N colonies was largely dependent on the colony areas and numbers of conidiophores formed in the colonies (see Table 1). Thus, these results reflected differences in lifelong conidial production among isolates and among different hosts. Overall, our data revealed that the number of progeny conidia dispersed from single KSP-7N colonies during its lifetime was almost the same as that from single KBP-01 (*B. graminis* f. sp. *hordei*) and KMP-6N (*Po. xanthii*) colonies described by Moriura et al. [34] and Suzuki et al. [36], respectively. Hirata [55] reported that a single barley powdery mildew colony on host leaves (chemically fixed samples), which was collected at various stages after inoculation, produced up to 2.0 × 10^5^ conidia during its lifetime. Thus, powdery mildew pathogens producing catenate conidia on conidiophores seem to consecutively release above 1.0 × 10^5^ conidia per a colony over their life under greenhouse conditions. To validate this working hypothesis, we proposed the utilisation of an electrostatic spore collector to calculate and compare the total number of all progeny conidia dispersed from single powdery mildew colonies under greenhouse conditions throughout their lifetime of different powdery mildew pathogens from the other or the same genus, possessing catenate conidia on conidiophores.

We assume that powdery mildew fungi continuously disperse progeny conidia produced on conidiophores in pathogen-free greenhouses throughout the day, in a manner unrelated to light intensity (day and night), for wide dispersal of powdery mildew diseases under the greenhouse conditions. The second finding of this study was vigorous conidial releases from KSP-7N colonies during the daytime; the colonies released few conidia during night-time (see Figure 3 and Table 3). Elucidation of the ecological characteristics of conidial released from KSP-7N colonies was impossible without the use of this electrostatic spore collector system. The mode of conidial release from KSP-7N colonies in this study was exactly the same as that from melon powdery mildew KMP-6N colonies, as previously described [36]. On the other hand, Suzuki et al. [54] previously described that KMP-6N formed a maximum of six conidia in chains on each conidiophore under greenhouse conditions, whereas KMP-6N formed over seven conidia in chains without forming full constrictions between the conidia in darkness. Moreover, KMP-6N actively dispersed progeny conidia from the colonies under greenhouse conditions, while few KMP-6N conidia were dispersed from the colonies in darkness. Consequently, in the present study, the conidial release of KSP-7N at night also seemed to be similar to the conidial release of KMP-6N in darkness described by Suzuki et al. [54] (see Figure 1F). The responses to light of strawberry and melon powdery mildew isolates, including the same *Podosphaera* genus, appeared to be clearly different from those of powdery mildew fungi, including the other genus. Actually, the numbers of conidia piled up on conidiophores were similar, regardless of the greenhouse conditions or growth chamber conditions, between tomato powdery mildew KTP-03 and KTP-04 (*Pseudoidium* genus) [28], Japanese Mallotus powdery mildew KMP-01 (*Oidium* genus) [28], red clover powdery mildew KRCP-4N (*Erysiphe* genus) [56], and barley powdery mildew KBP-01 (*Blumeria* genus) [31]. Furthermore, interestingly, conidial release from KSP-7N colonies largely reflected seasonal day length. The sunrise time in November and December was 1.5–2.0 h later than in May and July, while the sunset time in November and December was 1.5–2.0 h earlier than in May and July (see Figure 3 and Table 2). Suzuki et al. [54] reported that KMP-6N piled up ca. 40 conidia in chains on conidiophores, without releasing progeny conidia from conidiophores under growth chamber conditions or in darkness. Thus, the data obtained in the present study may also reflect the differences in light sensitivity of *Po. aphanis* var. *aphanis* KSP-7N, similar to *Po. xanthii* KMP-6N. In addition, the conidial release of KSP-7N will be largely affected by light intensity (photon flux density; PFD) as mentioned by Suzuki et al. [36]; conidial production gradually decreased with the light intensity. Surely, this study provides new information into the ecological characteristics of strawberry powdery mildew pathogens (*Po. aphanis* var. *aphanis*) on host plants. It is possible that KSP-7N conidiophores have light receptors that respond to light intensity in order to successfully constrict septa between conidial cells at the top of the conidiophores, similar to KMP-6N [36,48]. Thus, it is evident that light is essential for the release of conidia from conidiophores by KSP-7N.

Of the studies on strawberry powdery mildew pathogens (*Po. aphanis* (Wallroth) U. Braun and S. Takamatsu var. *aphanis*), to our knowledge this is the first to precisely count the total number of progeny conidia that released from individual colonies each month during their lifetime, directly determine the duration of conidial releases from single colonies, and clearly describe the differences in the number of progeny conidia collected from single colonies during the periods of the daytime and the night-time in a single day. Electrostatic techniques used in this study will be certainly useful to quantitatively analyse lifelong releases of progeny conidia from single powdery mildew colonies. We plan to apply the results obtained in the present study in future work, to gain insight into means for preventing the spread of powdery mildew fungi infection of strawberry.

## 4. Materials and Methods

### 4.1. Plant Materials and Cultivation

Seeds of strawberry (*Fragaria* × *ananassa* Duchesne cv. Sagahonoka) were obtained from the Nara Prefecture Agricultural Research and Development Center (Nara, Japan). The strawberry seeds were stored in a refrigerator at 4 °C for 7 days, and then were placed on wet filter papers inside Petri dishes. The seeds germinated for 14 to 16 days under conditions controlled at 22 ± 1 °C, 60 to 70% relative humidity (RH), and continuous illumination of 59.5 μmoL m^−2^ s^−1^. Cotyledonal seedlings were then placed on polyurethane cubic sponge supports (3 cm × 3 cm × 3 cm). The sponge supports and cotyledonal seedlings were inserted into 30-mL cylindrical plastic containers (diameter, 3 cm; length, 5 cm) containing 20 mL of liquid fertiliser (Kyowa, Osaka, Japan) and incubated for 60 days in an environment controlled at 22 ± 1 °C, 60 to 70% RH, illumination of 210 to 260 μmoL m^−2^ s^−1^, and photoperiods of 14 h light/10 h dark, as previously described by Iwasaki et al. [14]. Light intensity was measured using a LI-250A light meter (LI-COR, Tokyo, Japan) fitted with a quantum sensor that measures photosynthetically active radiation (400–700 nm).

**Figure 4:**
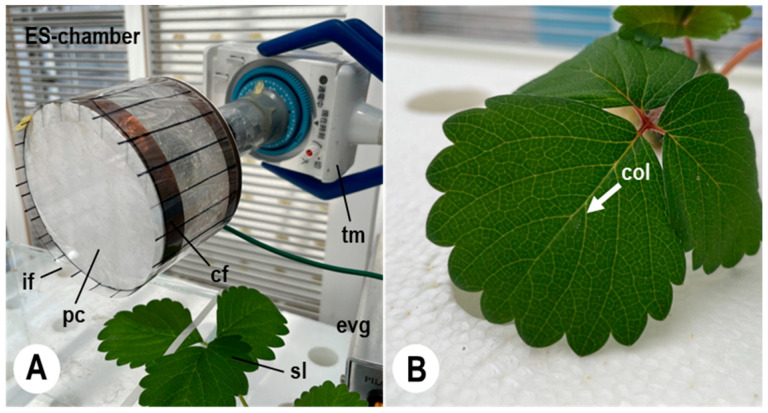
Electrostatic system for consecutively collecting progeny conidia dispersed from single colonies of *Po. aphanis* var. *aphanis* KSP-7N on true leaves of living strawberry seedlings. (**A**) Electrostatic spore collector used for consecutively collecting progeny conidia. The electrostatic spore collector (a rotary drum) consists of a round insulated plastic container (pc), a copper film (cf), a transparent insulator film (if), a timer mechanism (tm), and an electrostatic voltage generator (evg). The collector and seedling with a single KSP-7N colony on a strawberry leaf (sl) were prepared in an electrostatic screen chamber (ES-chamber) installed in the pathogen-free greenhouse. (**B**) Strawberry seedling grown hydroponically in a greenhouse, with a single 5-day-old KSP-7N colony (col) on the leaf.

The 60-day-old healthy seedlings (cv. Sagahonoka) were transferred to a polystyrene plate (61.5 cm × 60.5 cm × 3.0 cm) floating in hydroponic solution, in a hydroponic culture trough (67.0 cm × 65.5 cm × 21.0 cm) (Home Hyponica 303; Kyowa, Osaka, Japan) on a cultivating table (100 cm high) installed in a pathogen-free greenhouse (10.0 m × 6.0 m; 22 ± 1 °C) [33]. The seedlings were used for maintaining strawberry powdery mildew fungi and collecting all progeny conidia from a single colony of the powdery mildew isolate with an electrostatic spore collector (a rotary drum with insulator films wound around it), as shown in Figure 4A.

### 4.2. Fungal Materials, Inoculation and Incubation

A strawberry powdery mildew isolate (*Po. aphanis* (Wallroth) U. Braun and S. Takamatsu var. *aphanis* KSP-7N) [14] was used in the present study. KSP-7N conidia were collected from conidiophores using a pencil-type electrostatic insulator probe consisting of an ebonite rod 7 cm in length, 4 mm in diameter, and 5 μm in tip diameter at the pointed tip [14,32,35]. The insulator probe mounted on a micromanipulator was placed near a KH-2700 high-fidelity digital microscope (KH-2700 DM; Hirox, Tokyo, Japan). The true leaves of three 60-day-old healthy strawberry seedlings (cv. Sagahonoka) were inoculated with the KSP-7N conidia, as previously mentioned [14]. The KSP-7N isolate was maintained for 14 days after inoculation of conidia in an electrostatic screen chamber (ES-chamber) installed in a greenhouse (10.0 m × 6.0 m; 22 ± 1 °C, 50 to 70% RH, and illumination of 190.6 to 400.4 μmoL m^−2^ s^−1^) [33] and in a growth chamber (LH-240N; Nippon Medical and Chemical Instruments, Osaka, Japan; 22 ± 1 °C, 60 to 70% RH, and continuous illumination of 22.2 μmoL m^−2^ s^−1^) [14]. The ES-chamber was an apparatus for preventing airborne pathogens from entering the chamber. Moreover, the KSP-7N isolate used in this study was preserved in the Herbarium Preservation Section (Department of Agriculture, Kindai University, Nara, Japan). The 60-day-old strawberry leaves (cv. Sagahonoka) were inoculated with single KSP-7N conidia using the insulator probe mentioned above, and the strawberry leaf with a single 5-day-old KSP-7N colony (fungal fleck) was selected for the experiments (Figure 4B).

### 4.3. Electrostatic Spore Collector

**Figure 5:**
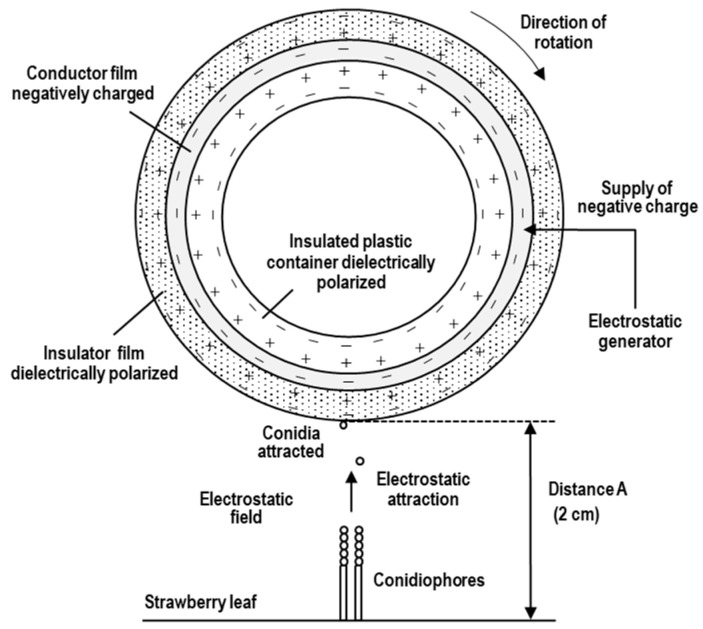
Diagram of the rotational electrostatic apparatus for collecting progeny conidia released from conidiophores in a single KSP-7N colony formed on a leaf of a strawberry seedling (cross-sectional view). A negative charge, which was produced from the electrostatic voltage generator, transferred to the conductor (copper) film. A positive “image charge” on the surface of the insulator film was induced by the negative charge on the conductor film. A negative surface charge on the opposite side of the insulator film was produced by dielectric polarisation. An electrostatic field was formed around the dielectrically polarised insulator film. Progeny conidia released from conidiophores were electrostatically attracted to the insulator film.

The electrostatic spore collector consisted of a round insulated plastic container (5 cm in height; 8 cm in diameter), a copper film (250 mm × 10 mm × 0.5 mm), a direct-current (DC) electrostatic voltage generator (HVA 10K202PA; Max Electronics, Tokyo, Japan), a transparent collector (insulator) film (260 mm × 60 mm × 0.5 mm) made with polypropylene (Hapila, Tokyo, Japan), and a timer mechanism (WH3311; Matsushita Electric Works, Osaka, Japan) (Figure 4A). The copper film was connected to the electric line from the electrostatic voltage generator. Electric current was supplied from the voltage generator to the copper film. The outer insulator film, negatively polarised and charged with static electricity of 5.2 × 10^−1^ nC, was placed ca. 2 cm from the single KSP-7N colony on a strawberry leaf for collecting all progeny conidia released, as previously mentioned [36]. An electrostatic field was produced by the negative charge on the outer surface of the electrified insulator film, and an attractive force for trapping progeny conidia that enter into the electrostatic field was created, as shown in Figure 5 [33,34,36]. The insulator film was removed from the device every 24 h to complete a rotation at the conidial collection site, and then a new insulator film was wound around the copper film of the rotary drum. The number of all progeny conidia collected from six individual 5-day-old KSP-7N colonies on each strawberry leaf with the insulator film was counted with a KH-2700 DM instrument.

### 4.4. Electrostatically Activated Insulator Film

The potential, which was supplied from the electrostatic voltage generator to the conductor film, dielectrically polarised the transparent insulator film (negatively charged on the conidium-collection side, and positively charged on the conductor film side as shown in Figure 5). The potential of the conductor film was controlled by the voltage generator, and an electrostatic field meter (FMX-002; Simco, Kobe, Japan) was used for measuring the potential difference (kV) between the insulator surface and ground level (the voltage). The probe (50 μm in tip diameter) of the coulometer (NK-1001; Kasuga Denki, Kanagawa, Japan) was used for measuring the surface electrostatic charge (nanocoulombs, nC) of the insulator film.

### 4.5. Continuous Collection of Progeny Conidia Dispersed from Single KSP-7N Colonies

Experiments were carried out to count the total number of progeny conidia dispersed from a single strawberry powdery mildew colony over a 35–45-day period after inoculation. KSP-7N conidia were collected from conidiophores that had formed in the fungal colonies under the greenhouse conditions using the electrostatic insulator probe [35] and were then transferred onto true leaves of six 60-day-old strawberry seedlings (cv. Sagahonoka), as mentioned above [14]. The KSP-7N-inoculated strawberry seedlings were grown for 5 days in an ES-chamber (22 ± 1 °C; 50–70% RH) [33], and the strawberry seedling with a single 5-day-old KSP-7N colony on a leaf (Figure 4B) was then placed under the electrostatic spore collector (rotary drum) (Figure 4A). The spore collector was consecutively operated for 30–41 days. The intervals between consecutive conidial releases were timed, and the collection procedure was repeated until conidia were no longer collected. The insulator film was consecutively charged at 5.2 × 10^−1^ nC on a single KSP-7N colony until being replaced by the next insulator film (film change duration of 30 s). A total of 30–41 films was used during each collection experiment. The number of progeny conidia deposited on each film was counted every 5 h after collection with the KH-2700 DM instrument. The number of progeny conidia collected per hour was estimated by pooling the counts for each 1 h interval. Finally, all conidia collected from a targeted single KSP-7N colony for a lifetime were counted. In the present study, we selected particular months relating to the seasons of occurrence of strawberry powdery mildew diseases for collecting all progeny conidia from single KSP-7N colonies with the methods mentioned above, and to confirm relationships between conidial releases from the strawberry powdery mildew colonies and photoperiods. Therefore, the experiment was conducted on just one colony on one leaf for each month (in February, May, July, October, November, and December in 2021), respectively.

### 4.6. Germination Rates of Conidia Collected with an Electrostatic Spore Collector

Germination rates of the KSP-7N conidia collected with the spore collector were tested. A strawberry seedling (cv. Sagahonoka) with a single KSP-7N colony was placed under the apparatus for each of the six months. The apparatus was operated as mentioned above. The 1st, 6th, 11th, 16th, and 21st (corresponding to 5-day-old, 10-day-old, 15-day-old, 20-day-old, and 25-day-old colonies after inoculation, respectively) insulator films were immediately removed after collection, placed in a humid box of 95–99% RH, and incubated at 22 ± 1 °C for 24 h. The number of KSP-7N conidia germinated was counted with the KH-2700 DM instrument. Data were presented as the mean and SD of six replicates (for each collection time in 20 February, 20 May, 20 July, 20 October, 20 November, and 20 December).

### 4.7. Observation of Single KSP-7N Colonies with Digital and Light Microscopes

The conidiophores formed in a KSP-7N colony on a strawberry leaf, and the progeny conidia attracted to the insulator film, were viewed with the MX-5030RZII objective zoom lens (250 magnifications) of the KH-2700 DM instrument. The zoom lens was focused on the side of both the film and the leaf. Morphological images of the conidia and conidiophores were digitally obtained with the one-half-inch interline transfer charge-coupled device camera of the KH-2700 DM instrument. Digital micrographs were analysed with Adobe Photoshop image-processing software (ver. 5.0; Adobe Systems, San Jose, CA, USA).

A single colony on a leaf for each month was used for each collection of progeny conidia, as mentioned above. After the final conidial collection, leaf segments (size; ca. 2 cm × 2 cm) were cut from the KSP-7N-infected strawberry seedlings. The leaf segments were fixed, and then leaf colour was removed by boiling in an alcoholic lactophenol solution including glycerol (10 mL), phenol (10 mL), lactic acid (10 mL), distilled water (10 mL) and 99.8% ethanol (40 mL) for 1 to 2 min. After that, leaves were stained using 0.1% Aniline Blue (Nacalai Tesque, Tokyo, Japan) dissolved in distilled water, as previously mentioned [57]. Subsequently, the stained KSP-7N colonies were observed under a BX-60 light microscope (BX-60 LM; Olympus, Tokyo, Japan) and photographed with a digital camera (EOS KISSX6i; Canon, Tokyo, Japan) mounted on the light microscope. The total number of conidiophores in the six individual KSP-7N colonies was calculated; moreover, these colony areas were determined using ImageJ software (NIH, Bethesda, MD, USA). Data were presented as the mean of six replicates.

### 4.8. Statistical Analysis with Tukey’s Test

Experimental data were presented as means and standard deviations (SD). Tukey’s test was performed using EZR software version 1.54 (Jichi Medical University, Saitama, Japan) to identify significant differences among the number of progeny conidia released from individual 20-day-old KSP-7N colonies in a single day during the daytime versus at night-time for each month.

## 5. Conclusions

All progeny conidia released from single colonies of the KSP-7N isolate over its lifetime were consecutively collected under greenhouse conditions using an electrostatic rotational spore collector (a rotary drum). This study clarified the number of conidia released over the lifetime from single KSP-7N colonies in each season (February, May, July, October, November, and December 2021). More importantly, this study demonstrated that the number of progeny conidia collected from the single KSP-7N colonies in the night-time was fewer than that released at daytime, and also that the release times of conidia from the single colonies were longer in spring and summer than autumn and winter. Conidial release from KSP-7N colonies was clearly affected by day length and light intensity, as indicated by the results obtained using our electrostatic spore collection system. Overall, electrostatic and digital microscopic techniques are useful for elucidating the ecological characteristics of conidial release from single powdery mildew colonies onto host plants under greenhouse conditions. As next steps, molecular elucidation of the light reaction for powdery mildew conidiophores is necessary to suppress the spread of infection of strawberry under greenhouse conditions.

## Figures and Tables

**Table 1:** Development of individual colonies of *Podosphaera aphanis* KSP-7N on leaves of strawberry seedlings during the period of conidiation by conidiophores, assessed by direct counting of progeny conidia consecutively trapped on insulator films electrostatically activated.

Colony *	Colony Area (cm^2^)	Number of Conidiophores in a Single Colony	Duration of Conidial Secession (day)	Total Conidia Collected
A	0.6	574	38	28,910
B	1.1	1034	34	103,208
C	0.6	594	34	34,674
D	1.0	957	39	98,504
E	0.5	498	29	56,117
F	0.7	650	28	83,058
Means	0.8	718	34	67,412

* Refer to Figure 2 for individual colonies (A–F).

**Table 2:** Daytime and night-time periods at the experiment location (greenhouses of Kindai University, Nara, Japan) for each month.

Date	Times of Sunrise	Times of Sunset	Periods of Daytime	Periods of Night-Time
20 February 2021	6:37 AM	5:44 PM	11 h 07 min	12 h 53 min
20 May 2021	4:50 AM	6:56 PM	14 h 06 min	9 h 54 min
20 July 2021	4:58 AM	7:08 PM	14 h 10 min	9 h 50 min
20 October 2021	6:06 AM	5:16 PM	11 h 10 min	12 h 50 min
20 November 2021	6:35 AM	4:49 PM	10 h 14 min	13 h 46 min
20 December 2021	6:59 AM	4:49 PM	9 h 50 min	14 h 10 min

Times of sunrise (white arrows) and sunset (black arrows) for each month are shown in Figure 3.

**Table 3:** The total number of progeny conidia collected from individual 20-day-old KSP-7N colonies in a single day during the periods of the daytime and the night-time for each month.

Date	The Number of Conidia Collected from a Single Colony at Periods of Daytime *	The Number of Conidia Collected from a Single Colony at Periods of Night-Time *
20 February 2021	1764	27
20 May 2021	7925	189
20 July 2021	2438	17
20 October 2021	6106	216
20 November 2021	2606	383
20 December 2021	2606	403
Means ± SD	3794.8 ± 2579.0 a	205.8 ± 166.3 b

* Daytime and night-time periods each month were represented in Table 2. Different letters (a, b) indicated significant differences (*p* < 0.05, Tukey’s test).

## Data Availability

Data sets analysed during the current study are available from the current author on reasonable request.
